# Definitions and Risk Factors for Drug-Resistant Epilepsy in an Adult Cohort

**DOI:** 10.3389/fneur.2021.777888

**Published:** 2021-12-13

**Authors:** Alyssa Denton, Lilian Thorpe, Alexandra Carter, Adriana Angarita-Fonseca, Karen Waterhouse, Lizbeth Hernandez Ronquillo

**Affiliations:** ^1^College of Medicine, University of Saskatchewan, Saskatoon, SK, Canada; ^2^Community Health and Epidemiology, University of Saskatchewan, Saskatoon, SK, Canada; ^3^Division of Neurology, Department of Medicine, University of Saskatchewan, Saskatoon, SK, Canada; ^4^Saskatchewan Epilepsy Program, Department of Medicine, Division of Neurology, University of Saskatchewan, Saskatoon, SK, Canada; ^5^Universite du Quebec en Abitibi-Temiscamingue, Rouyn Noranda, QC, Canada; ^6^Centre de Recherche du Centre Hospitalier del l'Universite de Montreal, Montreal, QC, Canada; ^7^Universidad de Santander, Bucaramanga, Colombia

**Keywords:** epilepsy, drug-resistant, new-onset, risk factors, uncontrolled seizures, adults, anti-seizure medication

## Abstract

**Background:** Less than one-third of people with epilepsy will develop drug-resistant epilepsy (DRE). Establishing the prognosis of each unique epilepsy case is an important part of evaluation and treatment.Most studies on DRE prognosis have been based on a pooled, heterogeneous group, including children, adults, and older adults, in the absence of clear recognition and control of important confounders, such as age group. Furthermore, previous studies were done before the 2010 definition of DRE by the International League Against Epilepsy (ILAE), so data based on the current definitions have not been entirely elucidated. This study aimed to explore the difference between 3 definitions of DRE and clinical predictors of DRE in adults and older adults.

**Methods:** Patients with a new diagnosis of epilepsy ascertained at a Single Seizure Clinic (SSC) in Saskatchewan, Canada were included if they had at least 1 year of follow-up. The first study outcome was the diagnosis of DRE epilepsy at follow-up using the 2010 ILAE definition. This was compared with 2 alternative definitions of DRE by Kwan and Brodie and Camfield and Camfield. Finally, risk factors were analyzed using the ILAE definition.

**Results:** In total, 95 patients with a new diagnosis of epilepsy and a median follow-up of 24 months were included. The median age of patients at the diagnosis of epilepsy was 33 years, and 51% were men. In the cohort, 32% of patients were diagnosed with DRE by the Kwan and Brodie definition, 10% by Camfield and Camfield definition, and 15% by the ILAE definition by the end of follow-up. The only statistically significant risk factor for DRE development was the failure to respond to the first anti-seizure medication (ASM).

**Conclusion:** There were important differences in the percentage of patients diagnosed with DRE when using 3 concurrent definitions. However, the use of the ILAE definition appeared to be the most consistent through an extended follow-up. Finally, failure to respond to the first ASM was the sole significant risk factor for DRE in the cohort after considering the age group.

## Introduction

Epilepsy is a devastating neurological disease associated with varying degrees of physical, mental, and social suffering. The seizure disorder can develop for a wide variety of reasons at any age, with 8% of the population experiencing a single seizure in their lifetime and 2% of those going on to develop epilepsy (1). Risk factors for developing epilepsy vary with age and specific etiologies. Such characteristics affect the course of the disease and are therefore crucial to the treatment and prognosis. However, with epilepsy research historically focusing on pediatric populations, new-onset epilepsy (NOE) in the adult population, namely, prognosis, has been incompletely examined.

The incidence of epilepsy in developed nations is 44/100,000 person-years (2–5), with the incidence of NOE being slightly higher in men (2, 3, 6, 7). The rate of epilepsy is highest in the first year of life; although, the percentage of childhood-onset epilepsy is decreasing with the aging population (8). As people live longer, the age-related incidence rates and associated etiologies have also shifted accordingly, resulting in a greater interest in this segment of the population.

Studies that spanned all age groups have concluded that focal epilepsy and focal onset seizures are more common, with the majority having an unknown etiology (2, 3, 6). The number of unknown etiologies found is likely due to both the analytic criteria used and the available technology during this time. Studies have shown that children with a known etiology had predominantly congenital abnormalities related to epilepsy; whereas in adults the more common etiologies included cerebrovascular disease, central nervous system (CNS) infection, CNS tumor, head trauma, and the congenital abnormalities seen in the pediatric population (2, 3, 6).

Unfortunately, drug-resistant epilepsy (DRE) can present at any age across the spectrum of patients with newly diagnosed epilepsy. Patients with DRE experience decreased quality of life (QOL) (9), significant medical, social, and financial burden, and increased mortality (10). Thus, the early identification of risk factors in adult patients who develop DRE will allow physicians a timelier identification for planned management and treatment, and for instance, allow for earlier referral for pre-surgical evaluation.

Recent studies have concluded that between 23 and 31% of epilepsy cases do not respond adequately to anti-seizure medication (ASM) (7, 11–13). However, DRE has been found to range from 15 to 60% depending on the definition criteria, cohort selection, and sample size. Also, most studies on DRE prognosis have been based on a pooled, heterogeneous group, including children, adults, and older adults, in the absence of clear recognition and control of important confounders, such as age group. In addition, it must also be remembered that these studies were conducted prior to the publication of the 2010 accepted definition of DRE by the International League Against Epilepsy (ILAE) (14). Lastly, available studies do not provide a comparison between different definitions of DRE in the same population.

In this study, we present a prospective cohort study of adults with NOE who were followed in our epilepsy clinic to accurately and precisely identify differences between 3 definitions of DRE and to identify risk factors associated with developing DRE, as defined by the ILAE (14).

## Methods

### Study Population

We identified retrospectively all adults who were referred to the Single Seizure Clinic (SSC) at the Royal University Hospital (RUH) in Saskatoon, Saskatchewan, between November 2011 and January 2018. Those included required a diagnosis of epilepsy at or after their initial appointment. Participants had to be 17 years or older at diagnosis of epilepsy. Included patients could not have a previous history, diagnosis, or treatment of seizures. All patients must have had at least 1 year of follow-up with an epileptologist.

### Diagnosis and Follow-Up

The SSC was opened in 2011 and is dedicated to the assessment and management of patients >17 years old referred from other medical professionals, primarily general practitioners, and emergency room physicians, after a first event suspicious for seizures. The clinic receives referrals from across the province of Saskatchewan, which has a population of approximately 1.1 million people. All patients assessed in the clinic receive a standard electroencephalogram (EEG) prior to the consultation in the SSC. Other assessments may include dedicated neuroimaging, 24-ambulatory EEG, and sleep-deprived EEG studies if clinically relevant.

The 2005 operational definition of epilepsy was used to identify patients as follows: patients with 2 seizures (>24 h apart), patients identified after 1 seizure with a risk of recurrence similar to that of patients after their second seizure (≥60%), or patients whose presentation is in keeping with an epilepsy syndrome (15). After a diagnosis of epilepsy is made, patients are prescribed a suitable ASM taking into consideration the individual characteristics, such as seizure type, comorbidities, side effects, and drug interactions.

After the initial epilepsy diagnosis, patients were followed by 2 epileptologists every 2–6 months for the first year, and thereafter, every 12–18 months. Patients could request earlier follow-up if needed by corresponding with the administrative staff of the clinic at the epilepsy clinic. At each visit, clinical information, compliance, and response to ASM were recorded. Doses of ASM were adjusted by clinical circumstances, efficacy, and tolerability. An add-up approach of ASM was used for patients whose epilepsy remained uncontrolled. Patients with identified foci requiring possible resection (i.e., mesial temporal sclerosis, tumor, arteriovenous malformation, etc.) were referred for neuro-surgical consultation. Data were collected from pre-existing patient files from the SSC, RUH health records, and prospective follow-up by epileptologists with a customized data abstraction tool. All procedures were aligned with the standards for epidemiologic studies (16).

### Outcomes of Interest

There were 2 measured outcomes. The first outcome was the diagnosis of DRE based on ILAE criteria by the treating epileptologist and its comparison with 2 more DRE definitions. The second outcome was the clinical predictors of DRE.

The diagnosis of DRE by the epileptologist using the definition of ILAE was as follow:

After 2 years of follow-up, patients who have failed to sustain seizure freedom despite adequate trials of 2 tolerated and appropriately chosen ASM regimens were considered to be patients with DRE. Conversely, seizure freedom requires 12 months of seizure freedom or 2 times the prior seizure interval, whichever is longer (14).

The ILAE definition of DRE was then compared to 2 alternative definitions as follows:

1. Kwan and Brodie (17): At 1-year post-diagnosis, patients who had seizures were by definition considered to have refractory epilepsy. Seizure-free status was defined as the lack of seizures of any type for a minimum of 1 year while receiving the same dose of ASM or after successful withdrawal of ASM.
2. Camfield and Camfield (18): At 1-year post-diagnosis, patients with an average of 2 or more seizures in each 2-month period during the last year of observation, despite treatment with at least 3 ASMs as monotherapy or polytherapy.

The 2 alternative definitions were chosen based on their good inter and intra-observer agreement (19).

### Clinical Predictor Factors for DRE

Independent variables were chosen based on their clinical relevance in describing the cohort, their possible association with the outcome variable as a risk factor, and on a prior review of the literature and expert opinion. The updated ILAE classification guidelines were used to define epilepsy characteristics, seizure characteristics, and epilepsy syndromes (20). All etiologies were confirmed with appropriate testing.

### Statistical Analysis

Statistical analysis was completed with Statistical Package Social Science (SPSS) Version 27 (SPSS Inc. IBM Corp., Armonk, NY, USA) and Stata 16 (StataCorp. 2019. Stata Statistical Software: Release 16. StataCorp. LLC; College Station, TX, USA). Statistical significance was defined by an alpha level of 0.05 and 95% CIs. Life tables were used to determine the cumulative risk of DRE diagnosis. Univariate analyses on continuous prognostic factors were performed using Cox regression, and categorical prognostic factors were analyzed using the Kaplan–Meier and the log-rank methods. The models were formulated by systematically removing predictors that were not significant (*P* > 0.05) starting from a (full) model containing all the prognostic factors, (including factors that were non-significant in the univariate analysis, but which were nevertheless, clinically significant. The proportional hazard assumption for each predictor was tested using the tests of the non-zero slope developed by Therneau and Grambsch (21) and Schoenfeld partial residuals. Finally, we used a stratified Cox regression analysis.

### Ethical Considerations

This study was approved by the Biomedical Research Ethics Board at the University of Saskatchewan (Bio#12-039).

## Results

Between November 2011 and January 2018, there were 887 patients seen at the SSC. Of these, a total of 95 patients met inclusion criteria for the study. Most of the patients were referred by an emergency room (ER) physician (66.3%) or a family physician (FP) (16.8%). In total, 51% of patients were male with a median age at diagnosis of epilepsy of 33 years (interquartile range [IQR]: 22, 52; maxi: 82 years).

The median duration of follow-up was 24 months (IQR 12.0, 36, max: 72 months). During this follow-up period, 3 patients had epilepsy surgery and 2 patients were deceased. Baseline characteristics are shown in Table 1.

**Table 1:** Clinical characteristics of 95 patients with a new diagnosis of epilepsy according to DRE status based on the 2010 International League Against Epilepsy definition.

**Characteristic**	**No DRE** ***n* (%)**	**DRE** ***n* (%)**	***p*-value^**†**^**
	**81 (80)**	**14 (20)**	
Age at onset-yr. [median (‡IQR 25–75)]	36.0 (23.0–53.0)	26.5 (20.0–37.0)	0.426^*^
Age group at onset-yr.			
17–60	64 (79.0%)	2 (14.3%)	0.983
>61 (Ref)	17 (21.0%)	12 (85.7%)	
Sex			
Male	40 (49.4%)	9 (64.3%)	0.163
Female (Ref)	41 (50.6%)	5 (35.7%)	
Developmental delay	6 (7.4%)	1 (7.1%)	0.418
History of: Family with epilepsy	14 (17.3%)	3 (21.4%)	0.940
Febrile seizures	1 (1.2%)	0	<0.001
Epilepsy type^a^			
Focal	44 (54.3%)	7 (50.0%)	0.311
Generalized	32 (39.5%)	5 (35.7%)	
Unknown (Ref)	5 (6.2%)	2 (14.3%)	
Seizure type^b^			
Focal onset	40 (49.4%)	6 (42.9%)	0.727
Generalized onset	39 (48.1%)	8 (57.1%)	
Unknown onset	2 (2.5%)	0	
No lesional (CT or MRI)	30 (57.7%)	5 (55.6%)	0.775
Psychiatric comorbidity	32 (41.6%)	4 (30.8%)	0.420
Two or more foci	4 (7.3%)	0	0.312
Failure to first ASM	28 (34.6%)	13 (92.9%)	<0.001
Specific etiology:			
Cortical dysplasia	2 (2.5%)	1 (7.1% )	0.775
Cranial trauma	3 (3.7%)	1 (7.1% )	0.995
Brian tumor	7 (8.6%)	0	0.991
Stroke	4 (4.9%)	1 (7.1%)	0.931
Mesial Temporal sclerosis	3 (3.7%)	2 (14.3%)	0.576
Arteriovenous malformation	2 (2.5% )	0	0.996
Other	3 (3.7%)	0	0.994
Unknown (ref.)	57 (70.4%)	9 (64.3%)	

† *p-values for the comparison of risk factors for DRE are based on Kaplan-Meier and the log-rank methods. ‡ IQR, = inter-quartile range; DRE, drug-resistant epilepsy.*

* *p-value was obtained by univariate Cox regression. a: accordingly with ILAE 2017 Classification of Epilepsy type, and b: accordingly with ILAE 2017 classification of Seizure type*.

### Therapeutic Management at Epilepsy Diagnosis

Thirty patients were started on ASM prior to attending the SSC. Phenytoin was prescribed in 19 (63.3%) patients, levetiracetam was prescribed in 6 (20%) patients, lamotrigine was prescribed in 3 (10%) patients, while carbamazepine and clobazam were chosen for 1 patient each. When medications were prescribed by the attending neurologists at SSC, the most frequent ASM prescribed was lamotrigine (63.2%) followed by phenytoin (15.4%) and levetiracetam (10.8%). In the overall cohort, 50 patients were found to be well controlled on the first ASM with no adverse effects, and 1 patient was well controlled albeit with side effects. In total, 39 patients were considered to have failed the first ASM with no adverse effects. In total, five patients failed trials of first ASM with adverse effects (Table 2).

**Table 2:** Response to anti-seizure medication trials in the cohort *n* = 95.

**Trial**	**Response^**†**^**	**No. (%)**
Response to first ASM	1A (Seizure free no adverse effects) 1B (Seizure free with adverse effects) IIA (Treatment failure without adverse effects) IIB (Treatment failure with adverse effects)	50 (52.6%) 1 (1.1%) 39 (41.1%) 5 (5.3%)
Response to second ASM	1A (Seizure free no adverse effects) 1B (Seizure free with adverse effects) IIA (Treatment failure without adverse effects) IIB (Treatment failure with adverse effects)	23 (24.2%) 0 19 (20.0%) 1 (1.1%))
Response to 3 or more ASM	1A (Seizure free no adverse effects) 1B (Seizure free with adverse effects) IIA (Treatment failure without adverse effects) IIB (Treatment failure with adverse effects)	10 (10.5%) 0 11 (11.6%) 0

† *Based on the ILAE definition for DRE. ASM, anti-seizure medication; ILAE, the International League Against Epilepsy; DRE, drug-resistant epilepsy*.

### Percentage Diagnosed With DRE by Diagnostic Criteria Used

A total of 30 (31.6%) of patients were diagnosed with DRE by using the Kwan and Brodie definition. In total, 22 patients at 12 months met these criteria. Of those 22 patients, 6 were censored after the first year and 7 no longer met the criteria at later follow-up appointments. In total, 4 new patients met the criteria for DRE at 24 months, 3 patients at 36 months, and 1 patient at 48 months. At 60 months and 72 months, no further diagnosis of DRE was made by this definition. In total, 5 patients had inconsistent follow-ups; in that, they were lost to follow-up for 1–2 consecutive years within the 72 months of the study.

When applying the Camfield and Camfield definition of DRE, a total of 10 patients (10.5%) met the criteria at some point within the 72 months of follow-up, however, only 7 remained drug-resistant by this definition in the last year of follow-up. In total, 5 patients were diagnosed at the first 12 months, 4 patients at the 48 months, and 1 patient at 36 months of follow-up. Of the 5 patients who met the criteria for drug resistance in the first year, 3 were censored after the 12 months, while 2 patients no longer met the criteria at 48 months and 60 months, respectively. Of the 4 patients diagnosed at the 48-month mark, 2 were censored after 48 months, 1 remained drug-resistant through follow-up, and 1 was not considered drug-resistant at the final follow-up. The patient diagnosed at 48 months, in the context of being lost to follow-up for the first 36 months, remained drug-resistant for the following 36 months of follow-up. Interestingly, at the last follow-up, 3 patients who were not considered drug-resistant by this definition, in fact, met the criteria for drug resistance by the Kwan and Brodie Definition. This indicates that they were still having seizures, but that these seizures fell outside the more specific time frame put forth by the Camfield and Camfield definition.

Implementing the ILAE definition of DRE, 14 patients (14.7%) fulfilled the criteria. Of the 14 patients, 10 were diagnosed at 48 months, which is the first available point of assessment according to this definition. Of those 10, only 1 was not considered to have DRE by the end of follow-up, which corresponded to their 60 months of follow-up. In total, 1 patient was diagnosed at 36 months and remained DRE for the remainder of the follow-up. In total, 3 patients did not fulfill the ILAE criteria until 60 months; despite meeting criteria for DRE based upon the Kwan and Brodie definition as early as 12 months (Table 2).

### Follow-Up Based on ILAE Definition of DRE

Of the 14 patients who met the criteria for the ILAE definition of DRE at the end of follow-up, 10 were identified at the 24 months of follow-up, 1 at the 36 months, 0 at 48 months, and 3 at 60 months. Thus, in the cohort, the cumulative incidence estimated by a life table, which considers the losses during the follow-up, at 24, 36, and 60 months was 19, 22, and 61% of new cases of DRE, respectively, following the new diagnosis of epilepsy.

Three patients in the cohort of 95 patients were not evaluated for follow-up at 12 months but were later followed up at 24 months; these cases were not found to be drug-resistant in the follow-up time of this study. Therefore, we can reasonably assume they did not significantly alter the validity of the percentage of DRE diagnosed early and throughout the follow-up period.

### Impact of Baseline Risk Factors (Univariate Analysis)

**Figure 1:**
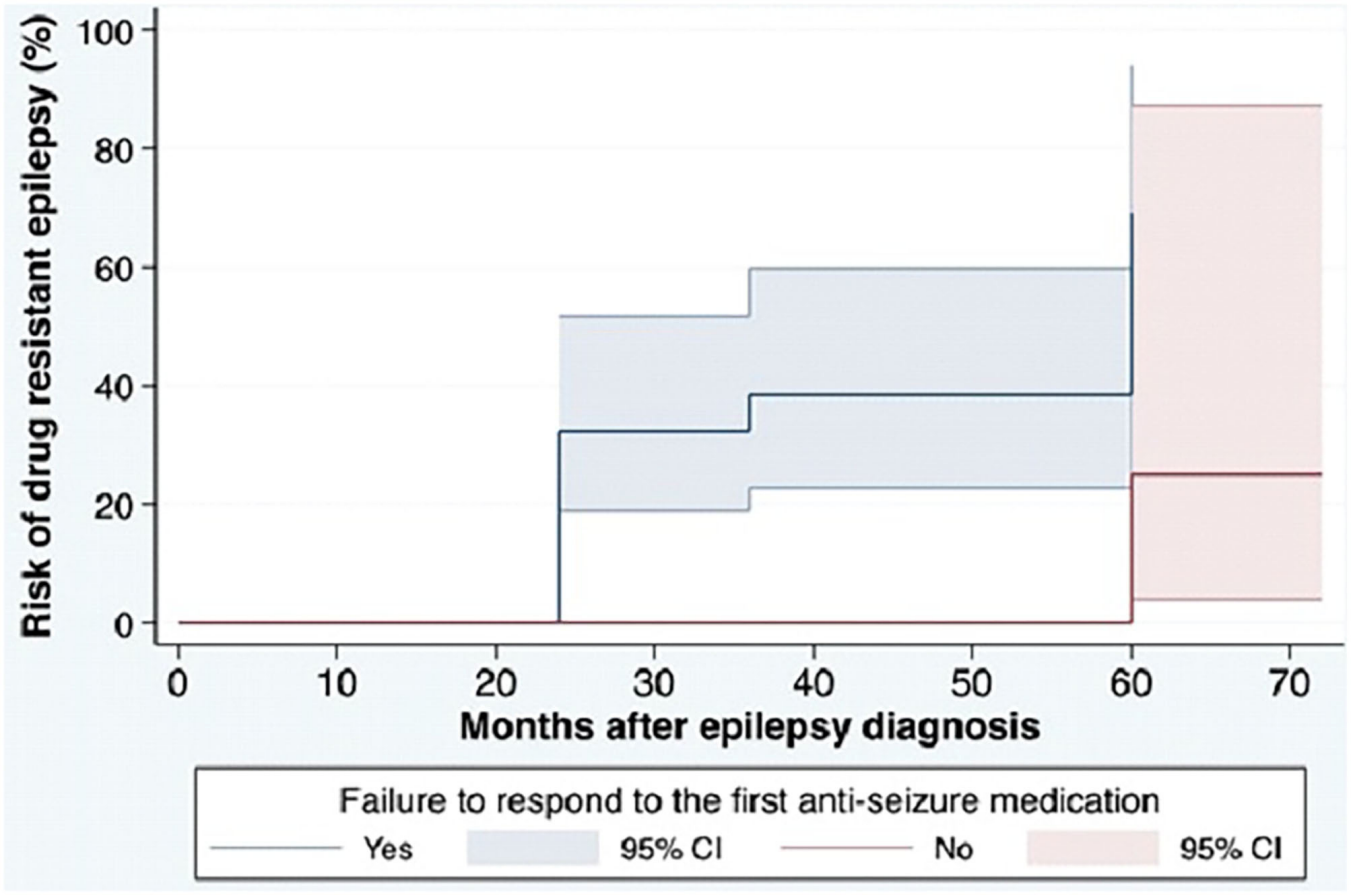
Cumulative incidence for DRE in the cohort based on ILAE definition. ILAE, the International League Against Epilepsy; ASM, anti-seizure medication; DRE, drug-resistant epilepsy. The cumulative risk for development of DRE in the cohort. The blue solid line shows the cumulative incidence of DRE among patients with failure to respond to the first ASM and the red solid line shows the cumulative incidence of DRE among patients without failure to respond to the first ASM. Colored areas show 95% CIs of the cumulative incidence.

Based on the ILAE definition, descriptive data for risk factors associated with DRE are shown in Table 1. Among the 95 patients in the cohort, patients with a diagnosis of DRE were younger than the no-DRE group with a median and IQR of 26.50 years (19.75, 40) vs. 36 years (22.5, 54) (*p*-value = 0.98). There were more males and more patients with developmental delay in the DRE group than in the non-DRE groups, although this did not meet statistical significance. There were no differences between the 2 groups regarding positive family history of epilepsy or a history of febrile seizures. Patients found to have psychiatric comorbidity were more frequent in the non-DRE group than the DRE group (*p* = 0.42). There were no statistical differences between the median number of seizures occurring at the onset of epilepsy between the 2 groups (*p* = 0.70). Failure of the first ASM was found to be statistically significant as a risk factor for developing DRE (Figure 1).

### Epilepsy Classification

Within the study cohort, focal epilepsy was identified in 51 patients (53.7%), 37 (38.9%) had generalized epilepsy and 7 had unknown epilepsy (7.4%). Seizure classification is shown in Table 1. A greater percentage of patients with an unknown epilepsy type was found in the DRE group; however, no significant differences were found between the 2 groups when considering seizure type, non-lesional epilepsy, multifocal epilepsy, or etiologies, such as cortical dysplasia, tumor, stroke, mesial temporal sclerosis, or arteriovenous malformation.

### Multivariate Analysis

The results of the stratified Cox regression analysis show that DRE, defined by the ILAE, occurred 13.5 times (95% CI: 1.71,105.56) more in patients who failed to respond to the first ASM, compared to those with a good response to the first ASM.

## Discussion

Cohort studies looking at epilepsy progression or NOE in adult populations are limited. The present study followed a cohort of patients, exclusively young and older adults, from the first diagnosis through several years of longitudinal follow-up to better characterize the evolution of the condition. Furthermore, this study followed an adult cohort with NOE looking specifically for the percent of epilepsy patients developing drug resistance. The rigorous inclusion/exclusion criteria for inclusion in the study allowed for a unique and well-defined exploration of the course of epilepsy and the various factors which play a role in the development of DRE. A significant strength of this study lies in our population-based referral and clinic model: all patients who have had a suspected first seizure in life are assessed within 3 months by a neurologist. Thus, the SSC model decreased the potential of referral bias for severe cases of epilepsy as cases assessed in the clinic have a broad spectrum of diagnoses, including syncope to psychogenic non-epileptic seizures, which are differential diagnoses of epilepsy. Nevertheless, increasing the SSC capacity.

### Therapeutic Management

Our findings show that there are differences in first prescribed ASM by ER/family physicians and neurologists at SSC. The preferred use of phenytoin among ER physicians and family physicians is in agreement with previous research in prescription trends among neurologists and non-neurologists (22). Also, non-neurologists incorporate the use of intravenous phenytoin as a first-line in the ER. Later, the neurologist assessed the appropriateness of any ASM prescribed prior to the SSC in agreement with the type of seizure/epilepsy presented. Moreover, the advantages and disadvantages of any medication were discussed with the patient, and the decision to carry on with a prescribed ASM before SSC was unanimously resolved. Despite that phenytoin is not the first line of treatment, most of the patients decided to continue using this medication out of concern for prolonged driving restrictions.

### Failure to ASM

In our study, the sole risk factor found for DRE development as defined by ILAE was the failure of the first ASM. This specific risk factor has been found in several previous studies in pediatric and adult populations (18, 23–29) and moreover, it has been specifically examined as a risk factor for DRE in pediatric epilepsy (17, 18, 29). The amount of evidence indicates that we should pay attention to the individual response to the first ASM and make timely referrals to epilepsy centers.

### Percent of DRE by Definition

Three validated definitions (19) were used to evaluate DRE across the 6 years of follow-up. Applying the ILAE definition of DRE, which is the most widely accepted definition, resulted in 14.7% of the cohort meeting criteria for DRE. The use of the Kwan and Brodie definition, which simply defines DRE as the continued occurrence of seizures, resulted in findings consistent with other studies, with a total of 31.6% DRE in the cohort (17, 30). The Camfield and Camfield definition, which uses more rigorous criteria for a DRE diagnosis, resulted in a much lower percentage of DRE [10.5% (Table 3)].

**Table 3:** Number of patients with DRE in each month of follow-up by definition.

	**Follow-up in months**					
**Definition**	**12** **(*n* = 92)**	**24** **(*n* = 64)**	**36** **(*n* = 40)**	**48** **(*n* = 17)**	**60** **(*n* = 12)**	**72** **(*n* = 2)**
ILAE (%)		10 (15.6)	1 (15.0)	0	3 (25.0)	0
Kwan & Brodie (%)	22 (23.9)	15 (23.8)	10 (25.0)	4 (23.5)	4 (33.3)	0
Camfield & Camfield (%)	5 (5.4)	5 (7.9)	3 (7.5)	1 (5.9)	1 (8.3)	0

*ILAE, International League Against Epilepsy; DRE, drug-resistant epilepsy*.

In another cohort of hospital-based epilepsy patients, DRE rates were found to be 34% using Kwan and Brodie criteria, 37% using Camfield and Camfield criteria, and 33% using the ILAE definition (19). We found no other studies evaluating the distribution of DRE by definitions among a cohort of adult patients with NOE from sub-specialty referral clinics, such as the SSC.

Both the Kwan and Brodie and the Camfield and Camfield definitions allowed for the evaluation of DRE after the first year of follow-up, which enabled us to then evaluate those patients by the ILAE definition of drug resistance in the later years to ensure they met the internationally accepted criteria. Those patients who met the diagnosis of Kwan and Brodie DRE in the first year but did not meet the ILAE definition in the following years of follow-up were not considered DRE for our analyses, which aimed to focus on the ILAE definition. Of the 22 patients diagnosed with DRE in the first year by either the Kwan and Brodie or the Camfield and Camfield definitions, 8 patients (36.4%) met the ILAE standards for DRE in the following year of follow-up, 1 patient (4.5%) met the ILAE in the fifth year of follow-up, 6 patients (27.3%) were censored after year 1, and 7 patients (31.8%) never meet the ILAE criteria for DRE. There were 6 patients (6.3%) in the entire cohort who met all 3 definition criteria at the same time at some point in their follow-up.

The first year after diagnosis presents an important opportunity to evaluate the progression and prognosis of NOE, which is reflected by the high percentage of DRE diagnosed at the 12-month follow-up point in our study. Those patients who had only 1 year of follow-up and met both the Kwan and Brodie and Camfield and Camfield DRE diagnoses can be considered as resistant to ASMs, due to the broad encompassing nature of the 2 definitions together. These highlight potential limitations in the ILAE definition which identifies patients later in the course of their illness, which may delay more intensive management plans. However, those patients identified as DRE by employing the ILAE definition continued to meet criteria for drug resistance throughout the course of the study, which may indicate that this is identifying a more truly “drug-resistant” population, rather than a population with more transient periods of drug resistance. Those identified by the Camfield and Camfield or the Kwan and Brodie definitions were more likely to go in and out of drug resistance, a pattern which is important to identify given that many treatments rely completely on a definitive definition of drug resistance. As time goes on, the treatments for DRE become more irreversible with each failed treatment, so having a reliable definition that considers possible variations in time is imperative to manage and ultimately outcomes of patients.

Worsening patient morale, depression, and QOL may be a potential pitfall in labeling a patient drug-resistant early in the course of their illness and may, as well, lead physicians to abandon further trials of ASM. This is another potential area of re-evaluation for the definition in future studies.

### The Overall Percent of DRE

The total percentage of DRE found in this adult cohort using the definition of the ILAE was approximately 15%. This is lower than other cohort studies, which were mainly done in epilepsy clinics. The majority of studies show rates higher than 30% of epilepsy cases who do not obtain remission via ASM regimens (5, 14, 19). It is important to note that most of these studies have been done in dedicated epilepsy clinics, which are naturally more referral biased toward complex and hard to treat cases of epilepsy, as opposed to a community-based population recruited from SSCs like ours. This means that 15% DRE may reflect a true population incidence of DRE, rather than the classically taught 30% incidence of DRE (19). This is in keeping with the recently published study by Sultana et al. They studied the incidence and prevalence of DRE by meta-analysis, examining the difference between studies of DRE performed in community-based populations vs. epilepsy clinic populations and found similar numbers of approximately 14% in community populations vs. 36% in clinic-based cohorts (31). Other community-based populations have reflected similar incidences of 15% (7). Studies, such as both older children and adults (9–93 years), indicate that 25–37% do not reach seizure freedom (17, 30). Our study utilized the internationally accepted ILAE definition for DRE, whereas the abovementioned studies had varying criteria for defining DRE, which were not necessarily validated or internationally accepted. Using the ILAE definition and the strict inclusion criteria increased the reliability and validity of our study.

### Percentage of DRE by Year of Follow-Up

The majority of patients who developed DRE according to the ILAE definition were diagnosed in the second year of follow-up (12/95). This Definition of DRE requires a certain set of criteria to be met at a given point in time. We studied patients through active follow-up processes which illustrated the dynamic nature of not only each individual's epilepsy but also the patterns of DRE presentation. When assessed year-to-year in follow-up for seizure frequency, medication adherence, and other comorbidities, we were able to track the progression of their condition over time. Many patients moved within categories from year-to-year. For example, 1 patient had a full 6 years of follow-up, during which they met the ILAE definition for the first time in year 5, but not in year 6. Potential areas of future research should include further exploration of patient predictors associated with such fluctuation in the recurrence of seizures. Other possible explanations of apparent intractability include ASM adherence and the presence of comorbid or un-identified non-epileptic seizures, which was not the case for this patient.

The ILAE definition utilizes 12 months as a timeline vs. 3 times the longest previous seizure-free interval, for seizure freedom and 2 years to diagnose DRE. These early periods post-epilepsy diagnoses are significant to patients in terms of overall QOL (32). Longer periods of intractability are a negative predictor for regaining seizure remission and are associated with worsening QOL (32, 33). By evaluating this time factor and DRE of a patient year-to-year, clinicians can better prognosticate their condition and QOL. Even 1 seizure in the previous 12 or 24 months has psychosocial consequences for a patient. In our cohort, 2 patients met the criteria for ILAE DRE but did not meet the same criteria at the last follow-up. Whether or not their QOLs in those years differed from their criteria-meeting counterparts could be a focus of future research in a year-to-year follow-up study. A future study could assess if and how QOL changes with the fluctuating states of DRE.

The focus of this analysis was the time elapsed between the onset of seizures and the development or diagnosis of DRE. The treatment history and duration of epilepsy are established risk factors for relapse over time and therefore are necessary points of observation in a follow-up study of this nature (34). In total, 2 prior studies, a pediatric and an all-ages prospective cohort study, indicated the latency period between onset of seizures and failure of the second ASM was 9.1 and 9.7 years, respectively (29, 35). Most patients in all ages study were <18 years of age, which is an important point to consider when applying such timelines to an adult cohort. The treatment process and timeline for adults with NOE are clinically different. As it is, the lapsed time for epilepsy surgery for adults in Saskatchewan is almost 20 years, similar to other centers which were 22.1 years (29, 35). Given that the definition of DRE utilizes a timeline of 2 years, there should be a much more rapid referral to tertiary epilepsy surgical centers in both our province and globally. Using the failure to first ASM as a point of referral to an Epilepsy Clinic for evaluation will expedite this and has been demonstrated by our study as being an important risk factor in the development of DRE. Having a better clinical understanding and prognosis of NOE at the onset of the condition of an individual, specifically those that are focal epilepsies, could ideally speed up the course of management and time waiting for life-altering, potentially curative surgery or other managements, such as vagal nerve stimulation.

### Strengths and Limitations of the Study

This is an important effort in terms of mapping the progression of NOE in an adult-specific cohort. Typically, epilepsy is recognized and diagnosed in children primarily and the research historically has focused on children. By identifying and following adults from the first seizure through the referral program set up at the SSC in Saskatchewan, we have the unique opportunity to diagnose, treat, and follow-up individual patients over their lifetime, but also have the ability to gain a greater understanding of the overall progression of epilepsy within this cohort.

The main strength of this study is the assessment of DRE through the use of different definitions and annual assessments. This has contributed to more accurate estimates of DRE in patients with NOE. Currently, there are mainly pediatric studies in epilepsy, so the information provided in this study will be useful for clinicians and researchers who mainly target adult patients with epilepsy. Also, the shorter follow-up time reduced the likelihood that changes in the efficacy of the available ASMs affected the changes seen over the years.

The cohort involves all adult patients referred and seen at the SSC who met NOE criteria. This has limitations because of reduced patient inclusion from the far northern and southern parts of the province where accessibility is a significant barrier. The SSC also would not have seen adults with NOE who were followed by a family physician or neurologist without referral to the SSC. This is less common though as our clinic is well established and more physicians and health providers know the procedures for a referral to the SSC in the province (referral of all suspected first seizures in adults to the SSC). However, we cannot reliably calculate what percentage of the Saskatchewan population we have captured with existing resources.

In terms of the patient cohort, the SSC patient screening tools at present do not ask for ethnicity, therefore, it is generally not included in patient charts. For this reason, we chose not to include ethnicity, or indigenous ancestry, as a risk factor in this study. This is a significant limitation given recent publications in Canada showing that indigenous persons are at 2 times the risk for developing epilepsy (36). It would then be reasonable to assume that it should be included as a risk factor for the progression of the condition as well, along with other ethnicities.

## Conclusion

The purpose of this study was to characterize the development of drug resistance in a purely adult cohort with NOE diagnosis from the SSC in Saskatchewan, Canada. The prospective study looked at 95 patients from approximately 6 years of possible follow-up between November 2011 and January 2018. The data collected allowed the analysis of risk factors and the possible relationships to the development of DRE over time through a Cox regression analysis.

Overall, the rate of DRE defined by the ILAE is lower than the rates seen in studies done in hospitals or epilepsy programs, primarily due to the clinic-based cohorts used in those studies. Our results of 15% drug resistance in adults reflect the true incidence of DRE in community-based populations.

The results of this study only identified the failure to respond to first ASM as a specific risk factor for the development of DRE. This is likely due to the specific inclusion criteria leading to small sample size. Failing the first ASM has important implications for the initial counseling and management of adult patients with NOE. In particular, primary physicians will have an evidence-based argument to encourage timely referral to a sub-specialty Epilepsy clinic.

## Data Availability Statement

The raw data supporting the conclusions of this article will be made available by the authors, without undue reservation.

## Ethics Statement

The studies involving human participants were reviewed and approved by Biomedical Research Ethics Board. Written informed consent for participation was not required for this study in accordance with the national legislation and the institutional requirements.

## Author Contributions

AD designed the original study, conducted the study, conducted some statistical analyses, and drafted the first version of the manuscript. LT and AC contributed to interpretation, drafting, and revising the manuscript. AA-F conducted the final statistical analysis. KW conducted the study, drafting, and revising the manuscript. LH is the senior author who designed the original study, conducted the first statistical analyses described herein, and drafted the original and final manuscript. All authors contributed to the article and approved the submitted version.

## Conflict of Interest

The authors declare that the research was conducted in the absence of any commercial or financial relationships that could be construed as a potential conflict of interest.

## Publisher's Note

All claims expressed in this article are solely those of the authors and do not necessarily represent those of their affiliated organizations, or those of the publisher, the editors and the reviewers. Any product that may be evaluated in this article, or claim that may be made by its manufacturer, is not guaranteed or endorsed by the publisher.
